# Supplementary material for: Identification of Resistance Determinants for a Promising Antileishmanial Oxaborole Series
Source: Microorganisms. 2021 Jun 29;9(7):1408. doi: 10.3390/microorganisms9071408 (PMC8305145; doi:10.3390/microorganisms9071408)
Supplement: Supplementary file 1 [file microorganisms-09-01408-s001.zip › microorganisms-1210853-supplementary.pdf]

SUPPLEMENTARY DATA

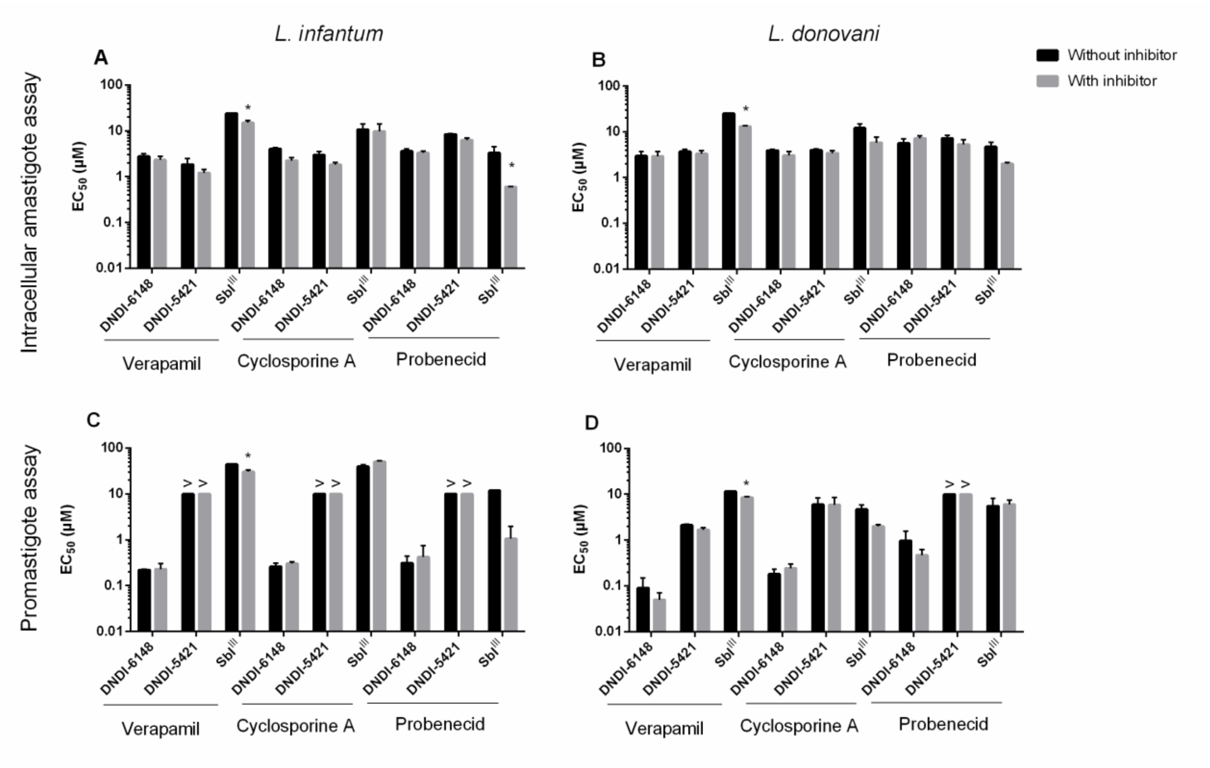

<u>Figure S1:</u> Effect of co-incubation of verapamil, cyclosporine A and probenecid with the oxaboroles for *L. infantum* (A&C) and *L. donovani* (B&D) in the intracellular amastigote (A&B) and promastigote (C&D) susceptibility assay. Results are based on two independent repeats run in duplicate and are expressed as the mean of the EC_{50} ± SEM (*p <0.05) (>: EC_{50} was higher than highest in-test concentration).

<u>Table S1</u>: Overview of the gene functions of the other highest enriched cosmids from the *L. infantum* Cos-Seq screen.

	Gene ID	Gene name	Max enrichment
Chromosome 35	LINF_350058100	Alpha-1-2-mannosyltransferase - putative	2071
	LINF_350058200	Homeobox associated leucine zipper - putative	2178
	LINF_350058300	WD domain - G-beta repeat - putative	2613
	LINF_350058400	Hypothetical protein - conserved	1738
	LINF_350058500	Lsm12 protein - putative	1123
	LINF_350058600	Hypothetical protein - conserved	921
	LINF_350058700	Ribosome-interacting GTPase 2 - putative	1009
	LINF_350058800	SAC3/GANP/Nin1/mts3/eIF-3 p25 family - putative	910
	LINF_350058900	Isopentenyl-diphosphatase delta-isomerase (type II) - putative	505

	LINF_350059000	Dynein heavy chain and region D6 of dynein motor/Anykrin repeats (3copies) - putative	824
	LINF_350059100	Amino acid permease - putative	724
Chromosome 34	LINF_340041100	Metallo-beta-lactamase superfamily - putative	231
	LINF_340041200	Cleavage and polyadenylation specificity factor - putative	770
	LINF_340041300	DNA topoisomerase IB - large subunit	616
	LINF_340041400	Hypothetical protein - conserved	662
	LINF_340041500	Hypothetical protein - conserved	480
	LINF_340041600	Amidinotransferase putative	502
	LINF_340041700	Hypothetical protein - conserved	648
	LINF_340041800	TPR repeat/Tetratricopeptide repeat - putative	532
	LINF_340041900	Ruvb-like 1 DNA helicase - putative	485
	LINF_340042000	Hypothetical protein - conserved	587
	LINF_340042100	Peroxisome biosynthesis protein-like protein	631
	LINF_340042700	Hypothetical protein - conserved	301
Chromosome 17	LINF_170020700	Cytidylyltransferase - putative	183
	LINF_170020800	Cytochrome b5-like Heme/Steroid binding domain containing protein – putative	160
	LINF_170020900	L-galactonolactone oxidase - putative	199
	LINF_170021000	NUC130/3NT domain/SDA1 - putative	121
	LINF_170021100	Hypothetical protein - conserved	129
	LINF_170021200	IQ calmodulin-binding motif containing protein - putative	159
	LINF_170021300	Myo-inositol-1(or 4)-monophosphatase 1 - putative	137
	LINF_170021400	Otubain cysteine peptidase - Clan CA - family C65 - putative	132
	LINF_170021500	Zinc-finger protein ZPR1 - putative	235
	LINF_170021600	Hypothetical protein - conserved	192
	LINF_170021800	Major Facilitator Superfamily - putative	209

Table S2: Genes encoded by the top 5 enriched regions of cosmids in DNDi-6148-resistant parasites following selection of the counter-screen cosmid library. Genes highlighted in green are rationalized to be the specific resistance determinants based on identification/location of barcodes. The two genomic fragments that were enriched also in the *L. infantum* Cos-Seq screening are highlighted on top.

	Gene ID	Gene name	RPKM
Chromosome 2	LdLV9.02.2.201140.1	ABC-2 family transporter protein/ABC transporter, putative	26.6
	LdBPK.02.2.000280.1	Soluble NSF attachment protein, SNAP, putative	126.5
	LdBPK.02.2.000290.1	3' exoribonuclease family, domain 1, putative	127.8
	LdBPK.02.2.000300.1	Glycosyltransferase family 10 (fucosyltransferase) C-term, putative	346.8
	LdBPK.02.2.000310.1	UAA transporter family, putative	460.2
	LdBPK.02.2.000320.1	hypothetical protein, conserved	490.9
	LdBPK.02.2.000330.1	Protein kinase domain/Protein tyrosine kinase, putative	449.1
	LdBPK.02.2.000340.1	PCI domain containing protein, putative	450.9
	LdBPK.02.2.000350.1	FtsJ-like methyltransferase, putative	539.9
	LdBPK.02.2.000360.1	Ubiquitin-conjugating enzyme, putative	593.3
	LdBPK.02.2.000380.1	hypothetical protein, conserved	436.0
	LdBPK.02.2.000390.1	hypothetical protein, conserved	407.3
	LdBPK.02.2.000400.1	hypothetical protein, conserved	178.9
	LdLV9.02.2.201150.1	Transmembrane amino acid transporter protein, putative	26.0
Chromosome 35	LdBPK.35.2.005180.1	50S ribosome-binding GTPase, putative	3.2
	LdBPK.35.2.005190.1	hypothetical protein, conserved	4.3
	LdBPK.35.2.005200.1	Putative methyltransferase, putative	4.4
	LdBPK.35.2.005210.1	hypothetical protein, conserved	32.9
	LdBPK.35.2.005220.1	ALG11 mannosyltransferase N-terminus/Glycosyl transferases group 1, putative	73.7
	LdBPK.35.2.005230.1	hypothetical protein, conserved	165.4
	LdBPK.35.2.005240.1	WD domain, G-beta repeat, putative	395.1
	LdBPK.35.2.005250.1	hypothetical protein, conserved	388.7
	LdBPK.35.2.005260.1	hypothetical protein, conserved	458.1
	LdBPK.35.2.005270.1	hypothetical protein, conserved	482.9
	LdBPK.35.2.005280.1	Ferrous iron transport protein B/50S ribosome-binding GTPase/C-terminal region of MMR_HSR1 domain/TGS domain containing protein, putative	460.5
	LdBPK.35.2.005290.1	SAC3/GANP/Nin1/mts3/eIF-3 p25 family, putative	408.8
	LdBPK.35.2.005300.1	FMN-dependent dehydrogenase, putative	436.0
	LdBPK.35.2.005310.1	Dynein heavy chain and region D6 of dynein motor/Ankyrin repeat, putative	407.0
	LdBPK.35.2.005320.1	Transmembrane amino acid transporter protein, putative	424.2
	LdBPK.35.2.005070.1	Tryptophan/tyrosine permease family/Transmembrane amino acid transporter protein, putative	434.7
	LdBPK.35.2.005060.1	Diacylglycerol kinase catalytic domain/Diacylglycerol kinase accessory domain containing protein, putative	462.1
	LdBPK.35.2.005050.1	hypothetical protein, conserved	452.3
	LdBPK.35.2.005080.1	SPRY domain/HECT-domain (ubiquitin-transferase), putative	176.7
Chromosome 21	LdBPK.21.2.001850.1	hypothetical protein, conserved	20.7
	LdBPK.21.2.001860.1	GMC oxidoreductase, putative	28.7
	LdBPK.21.2.001870.1	Protein kinase domain/Protein tyrosine kinase/Kinase-like, putative	48.1
	LdBPK.21.2.001880.1	Microtubule-binding protein MIP-T3, putative	109.1
	LdBPK.21.2.001890.1	hypothetical protein, conserved	173.6
	LdBPK.21.2.001900.1	Peroxidase, putative	171.3
	LdBPK.21.2.001910.1	hypothetical protein, conserved	163.6
	LdLV9.21.2.205030.1	hypothetical protein, conserved	192.9

	LdBPK.11.2.000660.1	hypothetical protein, conserved	383.8
	LdBPK.21.2.001940.1	F-box domain/F-box-like, putative	433.2
	LdBPK.21.2.001950.1	hypothetical protein, conserved	604.2
	LdBPK.21.2.001960.1	hypothetical protein, conserved	667.0
	LdBPK.21.2.001970.1	Ubiquitin-2 like Rad60 SUMO-like, putative	605.0
	LdBPK.21.2.001980.1	PPR repeat family, putative	532.2
	LdBPK.21.2.001990.1	EF hand/EF-hand domain pair, putative	476.0
	LdBPK.21.2.002000.1	hypothetical protein, conserved	448.0
	LdBPK.21.2.002010.1	Protein kinase domain/Protein tyrosine kinase, putative	381.4
	LdBPK.21.2.002020.1	5'-3' exonuclease, N-terminal resolvase-like domain/5'-3' exonuclease, C-terminal SAM fold, putative	421.2
	LdBPK.21.2.002030.1	hypothetical protein, conserved	398.5
	LdBPK.21.2.002040.1	hypothetical protein, conserved	399.1
	LdBPK.21.2.002050.1	Pumilio-family RNA binding repeat, putative	319.5
	LdBPK.21.2.002060.1	Mitochondrial calcium uniporter, putative	290.6
	LdBPK.21.2.002070.1	Proteasome subunit A N-terminal signature/Proteasome subunit, putative	238.8
	LdBPK.21.2.002080.1	hypothetical protein, conserved	105.8
	LdBPK.21.2.002090.1	Ribosomal protein L32, putative	56.8
**Chromosome 26**	LdBPK.26.2.002510.1	hypothetical protein, conserved	26.1
	LdBPK.26.2.002520.1	hypothetical protein, conserved	36.3
	LdBPK.26.2.002530.1	hypothetical protein, conserved	37.3
	LdBPK.26.2.002540.1	FYVE zinc finger/Protein tyrosine kinase/Protein kinase domain containing protein, putative	46.6
	LdBPK.26.2.002550.1	hypothetical protein, conserved	171.7
	LdLV9.26.2.206240.1	Calcineurin-like phosphoesterase, putative	202.7
	LdBPK.26.2.002570.1	hypothetical protein, conserved	387.8
	LdBPK.26.2.002580.1	hypothetical protein, conserved	585.8
	LdBPK.26.2.002590.1	ATP-grasp domain/D-ala D-ala ligase C-terminus/SET domain containing protein, putative	570.9
	LdBPK.26.2.002600.1	Protein tyrosine kinase/Protein kinase domain containing protein, putative	692.3
	LdBPK.26.2.002620.1	Paraquat-inducible protein A, putative	713.5
	LdBPK.26.2.002630.1	hypothetical protein, conserved	842.2
	LdBPK.26.2.002640.1	FAD binding domain/NAD(P)-binding Rossmann-like domain containing protein, putative	698.2
	LdBPK.26.2.002650.1	hypothetical protein, conserved	652.6
	LdBPK.26.2.002660.1	CSL zinc finger containing protein, putative	657.0
	LdBPK.26.2.002670.1	hypothetical protein, conserved	549.1
	LdBPK.26.2.002680.1	HECT-domain (ubiquitin-transferase), putative	425.4
	LdBPK.26.2.002690.1	hypothetical protein, conserved	201.0
	LdBPK.26.2.002700.1	ABC transporter transmembrane region/ABC transporter, putative	95.8
	LdBPK.26.2.002710.1	hypothetical protein, conserved	27.2
	LdBPK.26.2.002720.1	CAAX protease self-immunity, putative	0.8
**Chromosome 26**	LdBPK.26.2.000700.1	hypothetical protein, conserved	108.3
	LdBPK.26.2.000710.1	Regulator of chromosome condensation (RCC1) repeat, putative	229.7
	LdBPK.26.2.000720.1	Regulator of chromosome condensation (RCC1) repeat, putative	437.9

LdBPK.26.2.000730.1	RNA recognition motif. (a.k.a. RRM, RBD, or RNP domain)/RNA recognition motif (a.k.a. RRM, RBD, or RNP domain), putative	589.7
LdBPK.26.2.000740.1	hypothetical protein, conserved	560.7
LdBPK.26.2.000750.1	RNA recognition motif. (a.k.a. RRM, RBD, or RNP domain), putative	703.5
LdBPK.26.2.000760.1	hypothetical protein, conserved	826.5
LdLV9.26.2.206120.1	Glutathione peroxidase, putative	579.8
LdLV9.26.2.206130.1	Glutathione peroxidase, putative	817.8
LdBPK.26.2.000790.1	Aspartate-ammonia ligase, putative	978.2
LdBPK.26.2.000800.1	hypothetical protein, conserved	637.2
LdBPK.26.2.000810.1	hypothetical protein, conserved	655.4
LdBPK.26.2.000820.1	Endonuclease/Exonuclease/phosphatase family, putative	611.7
LdBPK.26.2.000830.1	hypothetical protein, conserved	505.4
LdLV9.26.2.206140.1	Ribosomal protein S9/S16, putative	392.6
LdLV9.26.2.206150.1	Ribosomal protein S9/S16, putative	316.1
LdBPK.26.2.000860.1	Hsp70 protein, putative	152.6
LdBPK.26.2.000880.1	hypothetical protein, conserved	68.0
LdBPK.26.2.000890.1	hypothetical protein, conserved	94.7
LdBPK.26.2.000900.1	hypothetical protein, conserved	68.0
LdBPK.26.2.000910.1	DnaJ domain/Domain of unknown function (DUF3395), putative	19.4
